# The Effect of Blood Flow-Restricted Low Resistance Training on Microvascular Circulation of Myocardium in Spontaneously Hypertensive Rats

**DOI:** 10.3389/fphys.2022.829718

**Published:** 2022-03-24

**Authors:** Zhaowen Tan, Yan Zhao, Yuchan Zheng, Ying Pan

**Affiliations:** ^1^ College of Sports Science, Nanjing Normal University, Nanjing, China; ^2^ Nanjing Sport Institute, Nanjing, China

**Keywords:** blood flow-restricted low resistance training, hypertension, myocardium, microvascular circulation, cardiac function, blood pressure

## Abstract

**Objective:** The purpose of this study was to explore the effect of blood flow-restricted low resistance training on microvascular rarefaction in the myocardium of spontaneously hypertensive rats (SHRs).

**Methods:** Four-week-old male SHRs were randomly divided into the following groups: Wistar-Kyoto (WKY), SHR control (SHR-SED), high-intensity resistance training (HIRT), low-intensity resistance training (LIRT), and blood flow-restricted low resistance training (BFRT). The exercise groups began to receive exercise intervention for 8 weeks at the age of 7 weeks. Blood pressure (BP), heart rate (HR), cardiac function, capillary density, and Vascular endothelial growth factor -Phosphatidylinositol 3-kinase-Protein kinase B-Endothelial nitric oxide synthetase (VEGF-Pi3k-Akt-eNOS) were assessed.

**Results:** 1) BP and HR of BFRT decreased significantly, Ejection fraction (EF) and Fraction shortening (FS) increased, and the effect of BFRT on lowering BP and HR was better than that of other groups (*p* < 0.05); 2) The expression of VEGF, VEGFR2, p-VEGFR2, Pi3k, Akt, p-Akt, eNOS and p-eNOS in the myocardium of the BFRT was significantly upregulated, and eNOS expression was significantly higher than other groups (*p* < 0 05); 3) the expression of VEGF in the blood of the BFRT was significantly upregulated, higher than SHR-SED, lower than HIRT (*p* < 0.05), and there was no significant difference between BFRT and LIRT(*p* > 0.05); 4) the capillary density in the myocardium of BFRT was significantly higher than other exercise groups (*p* < 0 05).

**Conclusion:** Blood flow-restricted low resistance training can activate the VEGF-Pi3k-Akt-eNOS pathway, upregulate the expression of VEGF in blood, improve microvascular rarefaction, and promote myocardial microvascular circulation, thereby improving cardiac function and lowering blood pressure, achieving the preventive effect of early hypertension.

## Introduction

Hypertension is a risk factor to cardiovascular disease that deserves attention. Biomechanical stress related to hemodynamic load caused by long-term hypertension gradually changes cardiovascular function, under the action of mechanical and humoral factors (Sharman et al., 2015). Cardiomyocytes transform into fibroblasts, while vascular endothelial cells undergo apoptosis and necrosis, which finally leads to vasodilation/contraction dysfunction and the formation of myocardial fibrosis, which also forces cardiac microvessels to contract to no blood perfusion, resulting in functional and structural sparsity of myocardial capillaries (Levy et al., 2001; Chantler and Frisbee, 2015; Vega et al., 2017), further increasing cardiac functional load (Vega et al., 2017; Scott et al., 2019). Exercise can increase the expression of nitric oxide synthase in cardiac endothelial cells, which has beneficial effects on cardiac tissue and its microvasculature (Duncker and Bache, 2008).

Activation of the classical Pi3k-Akt-eNOS pathway triggers the release of a large amount of eNOS that regulates disordered cardiomyocytes, improves the environment of fibrosis in the myocardium, and induces cardiomyocytes to grow normally (Farah et al., 2017; Yin et al., 2018; An et al., 2019; Jia et al., 2019). To maintain cardiac contractile function, at the same time, the activation of the Pi3k-Akt-eNOS pathway and the release of a large amount of NO stimulate the expression of VEGF and promote the growth of blood vessels in the myocardium (Moreno et al., 2017; Vega et al., 2017). The activation of the Pi3k-Akt-eNOS pathway and the regulation of the expression of VEGF improve the function of endothelial cells in the vascular myocardium, as well as stimulate capillary neovascularization and promote the occurrence of microvascular circulation, thereby regulating cardiovascular function (Tao et al., 2015; Farah et al., 2017).

Blood flow-restricted low resistance training is a novel resistance training method that results in short-term limitation of vascular blood flow of limbs using an adjustable pressure cuff, and combined with low-intensity resistance training, provides an alternative scheme for people who are not capable of engaging in high-intensity resistance training but need to increase muscle strength. This training program has been proven to be safe and feasible (Sato, 2005; Boeno et al., 2018). In addition, blood flow-restricted low resistance training can improve the cardiac function of elderly women and effectively lower the blood pressure of postmenopausal women (Ladlow et al., 2018; Naderi-boldaji et al., 2018; Raji-Amirhasani et al., 2018; Törpel et al., 2018; Yu et al., 2018). However, in terms of hypertension, studies on the mechanism of blood flow-restricted low resistance training to lower blood pressure and improve cardiac function are limited.

Therefore, for the rationality of grouping, this study referred to the grouping methods in BFR related studies (Picón et al., 2018; Raji-Amirhasani et al., 2018), compared BFRT with traditional resistance training, discussesed the effects of different training methods on blood pressure and cardiac function of spontaneously hypertensive rats and compare the effect of lowering blood pressure and the improvement of cardiac function; Analyzed the expression of VEGF-Pi3k-Akt-eNOS protein in the myocardium and microvascular rarefaction in the myocardium; To explore the possible mechanism to provide an exercise program for the early prevention of hypertension.

## Materials and Methods

### Animals

Sixty clean-grade male spontaneously hypertensive rats and fifteen clean-grade male Wistar-Kyoto rats (4 weeks old) weighing approximately 200 g were obtained from Qinglongshan Animal Feeding base in Jiangning District, Nanjing, China. Animals were housed in a 20–23 °C environment on a 12-h light-dark cycle with free access to water and food. Animal breeding was in full compliance with the relevant regulations of the Animal Ethics Committee of Nanjing Sport Institute of Physical Education. The study was approved by the Animal Experiment Ethical Inspection Form of Nanjing Sport Institute.

### Grouping of Rats

After 1 week of adaptive feeding, all rats were randomly divided into five groups: WKY (Wistar-Kyoto, *n* = 15), SHR-SED (SHR controls, *n* = 15), HIRT (high-intensity resistance training, *n* = 15), LIRT (low-intensity resistance training, *n* = 15), and BFRT (blood flow-restricted low resistance training, *n* = 15).

### Exercise Plan

The exercise groups trained on a ladder suitable for rats, with a total of 54 vertical steps with a 0.5-cm interval between steps. There was a rest platform at the top of the steps, which created a stable environment for animals to climb and rest. Before the maximum load test, all animals were accustomed to climbing for two consecutive weeks (adaptive ladder climbing practice, no load, 5 days a week, climbing 1 group every day, 15 times in each group). The test included an initial load that was 75% of the body weight, the load was attached to the base of the rat tail, and the load was gradually increased by 50 g during the subsequent climb. During formal training (the rats began formal training at the age of 7 weeks), the standardized value of the maximum load of each rat (the load/bodyweight of the last complete climb) was used for resistance training and adjusted according to the animal weight each week (Table 1).

**TABLE 1:** The maximum load of the exercise groups.

**Week**	**Maximum load (g)**		
	HIRT	LIRT	BFRT
1	222.7 ± 8.2	223.8 ± 1.5	223.6 ± 7.4
2	234.5 ± 7.7	236.4 ± 6.5	239.8 ± 9.7
3	252.1 ± 4.2	244.3 ± 3.1	254.4 ± 5.4
4	260.8 ± 1.4	242.7 ± 7.7	263.2 ± 3.6
5	346.5 ± 7.4	323.5 ± 7.2	344.3 ± 3.1
6	389.8 ± 2.1	359.7 ± 7.1	368.6 ± 7.4
7	443.2 ± 6.5	402.1 ± 9.7	414.3 ± 2.3
8	469.8 ± 7.4	420.1 ± 3.2	432.5 ± 4.4

**FIGURE 1:**
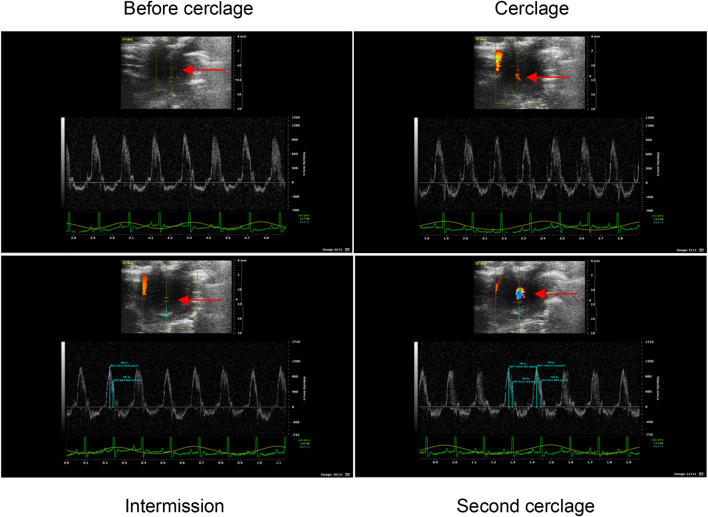
Ultrasonic detection of blood flow limitation in the right lower limb of BFRT group rats. The arrow marks the location of blood flow limitation of the right lower limb of BFRT group rats, and the red and blue bright spot at the mark is effective ischemia.

Blood flow-restricted low resistance training: 5 days a week; weeks 1–2: 30–40% of the maximum load; weeks 3–5: 40–50% of the maximum load; weeks 6–8: 40–60% of the maximum load. Simultaneous with climbing the ladder, the rubber band was used to bind the root of the thigh of the right lower limb of the rats to apply vascular blood flow limitation with resistance training. At 1-min intervals, blood flow restriction was lifted, and blood flow was reperfused. After rest, the rubber band was used to bind the site again. To better monitor the degree of blood flow limitation, small animal high-frequency color ultrasound (Visual Sonics Co., Toronto, Canada) was used to monitor blood flow in the BFRT rat group after cerclage, during intermission, and during the second cerclage to achieve 30–40% of the training needs of blood flow limitation (Tan et al., 2020) (Figure 1).

Low-intensity resistance training: 5 days a week; weeks 1–2: 30–40% of the maximum load; weeks 3–5: 40–50% of the maximum load; weeks 6–8: 40–60% of the maximum load.

High-intensity resistance training: 5 days a week; weeks 1–2: 50–60% of the maximum load; weeks 3–5: 60–70% of the maximum load; weeks 6–8: 70–80% of the maximum load (Table 2).

**TABLE 2:** Resistance training plan.

Week	Number of climbing ladders/day			Intermission time			Resistance (maximum load percentage)		
Adaptive training for a week									
	HIRT	LIRT	BFRT	HIRT	LIRT	BFRT	HIRT	LIRT	BFRT
1	15	15	15	1min	1min	1min	50%	35%	35%
2	15	15	15	1min	1min	1min	50%	35%	35%
3	15	15	15	1min	1min	1min	65%	45%	45%
4	15	15	15	1min	1min	1min	65%	45%	45%
5	15	15	15	1min	1min	1min	65%	45%	45%
6	15	15	15	1min	1min	1min	75%	55%	55%
7	15	15	15	1min	1min	1min	75%	55%	55%
8	15	15	15	1min	1min	1min	75%	55%	55%

### Measurement of Blood Pressure and Heart Rate

The caudal artery blood pressure of rats was measured noninvasively at the Jiangsu Medical Animal Experimental Center. During the test, the environment was quiet, warm, and appropriate, the rats needed to keep awake. The caudal artery blood pressure and heart rate of rats at rest were measured using an intelligent non-invasive blood pressure tester BP-2000 (Ruanlong Biological Co., Beijing, China). Each rat was measured continuously thrice, and the average value was taken. The measurement period was from 9:00 to 11:00 on the Saturday of the corresponding week (before training, after 8 weeks of training) (Fu et al., 2016).

### Echocardiography

The hearts of the rats were examined by echocardiography at the Jiangsu Medical Animal Experimental Center. After applying 2–3% isoflurane anesthesia to the rats, a small animal high-frequency color ultrasound (Visual Sonics) was used to evaluate cardiac function in the five groups, including ejection fraction (EF), and fraction shortening (FS); all rats were measured thrice. The measurement period was conducted from 9:00 to 11:00 on the Saturday of the corresponding week before and after 8 weeks of training (Naderi-boldaji et al., 2018).

### ELISA

After intraperitoneal injection of 10% chloral hydrate (0.5 ml/100 g) to rats, the abdominal fascia and other tissues were removed with a coarse gauze, and blood was collected from the inferior vena cava. The blood was left to stand at room temperature for 2 h, then centrifuged at 3,000 rpm for 20 min, the supernatant collected, and assessed by an ELISA detection kit (Jiangsu Kaiji Biotechnology Co.) following the manufacturer’s instructions. The level of vascular endothelial grown factor (VEGF) in serum was evaluated using an enzyme labeling instrument (Beckman Co., United States), and the index level was calculated according to the standard curve (Zhao et al., 2020).

### Western Blotting

After dissection, the hearts of rats were removed, and cleaned with normal saline, dried with filter paper, and cryopreserved at −80°C. The left ventricular tissues of rats were taken and ground into powder in liquid nitrogen, and then incubated with cell lytic solution for 30 min. Then, the solution was centrifuged at 10,000 rpm at 4°C for 5 min, then the supernatant was collected. The total protein concentration of the solution was determined using the BCA protein quantitative method. Equal amounts of protein (50 μg total protein/well) were separated on 8% SDS-polyacrylamide gels and transferred onto a PVDF membrane (Bio-Rad Laboratories, United States). The membranes were blocked with 3% non-fat milk solution in Tris-buffered saline (TBS) with 0.1% Tween 20 (TBS-T) for 1.5 h, then incubated overnight at 4°C with monoclonal primary antibodies against VEGF-Pi3k-Akt-eNOS and GAPDH (anti-VEGFA, 1:1,500; anti-VEGFR2, 1:1,000; anti-p-VEGFR2, 1:1,000, Cell Signaling, United States; anti-Pi3k, 1:1,000; anti-Akt, 1:1,000; anti-p-Akt, 1:2,000; anti-eNOS, 1:1,000; anti-p-eNOS, 1:1,000; anti-GAPDH, 1:10,000, ProteinTech Group, United States). After washing for 30 min (3 washes of 10 min) in TBS-T, the membranes were incubated with a polyclonal peroxidase-conjugated secondary antibody (anti-rabbit IgG-HRP, 1:1,000, ProteinTech Group) and incubated at room temperature for 1.5 h. Finally, enhanced chemiluminescence (ECL) was added, and the membranes were placed in a Bio-Rad Chemidoc XRS+(Bio-Rad, United States) for exposure, and grayscale was analyzed with the image analysis software Image LabTM (Da Costa Rebelo et al., 2012).

### Immunofluorescence

Rat left ventricular tissues were fixed with 4% paraformaldehyde, embedded in paraffin, cross-cut tissue, section thickness 5 μm. Fixed tissue sections were permeabilized with Triton (0.1%) for 10 min and blocked in PBS with 5% BSA for 1 h. Then, the samples were incubated with primary antibodies VEGF (1:800; ProteinTech Group, United States) overnight at 4°C. After three washes with PBS, the samples were incubated with fluorescent secondary antibodies Cy3-or FITC-conjugated IgG (1:400; ProteinTech Group, United States) for 1 h at room temperature, followed by 10 min of 4,6-diamino-2-phenylindole (DAPI) (Servicebio, China) staining for nuclei visualization, and the images were captured with a fluorescence microscope (NIKON ECLIPSE C1, Nikon, Japan). Image-pro plus 6.0 (Media Cybernetics, Inc, Rockville, MD, United States) was used to perform analysis of immunofluorescence signals (Cao et al., 2020).

### Immunohistochemical Assessments

Rat left ventricular tissues were fixed with 4% paraformaldehyde, embedded in paraffin, cross-cut tissue, section thickness 5 μm. The sections were deparaffinized by sequential washing with xylene and dehydrated in a graded series of ethanol, and then antigen repair and blocking were carried out. Primary antibody CD31 (1:100, Abcam, United Kingdom) diluted with phosphate buffered saline (PBS) was added dropwise, used as an angiogenesis marker to represent the capillary vessels in myocardium, overnight at 4 °C. The next day, secondary antibody was added and incubated at 37 °C for 0.5 h. After PBS washing, diaminobenzidine (DAB) chromogenic solution was added. Hematoxylin stained the nucleus, gradient ethanol dehydration, neutral gum sealing, and observed under light microscope. Capillaries were visualized in the myocardium as a brown precipitate. Five fields in the target zone at ×400 magnification were randomly selected for calculating microvessel density (MVD), and analyzing integral optical density (iod) value using Image Pro Plus 6.0 (Xu et al., 2017).

### Statistical Analysis

The statistical data were expressed as the mean ± SD, ANOVA was used for inter-group comparison, paired *t*-test was used to analyze difference within the group, Pearson correlation analysis was used to assess the correlation between two variables. Statistical significance was established at *p* ≤ 0.05. Image Lab was used to analyze the results of western blotting, and GraphPad Prism 8 was used to generate the statistical chart.

## Results

### Bodyweight, Hemodynamic Parameters and Cardiac Function

**FIGURE 2:**
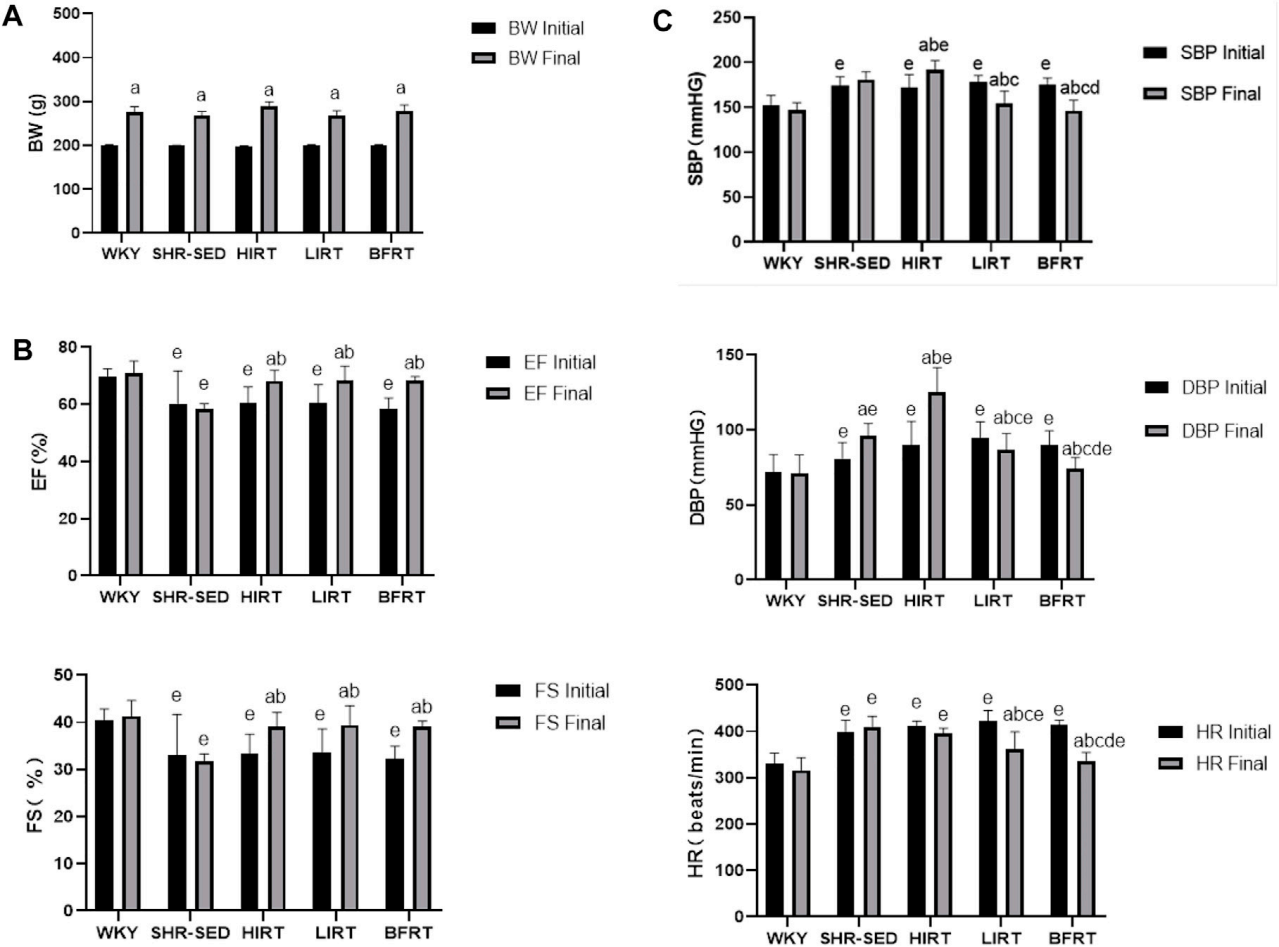
**(A)** body weight; **(B)** cardiac function; **(C)** hemodynamic parameters.

The results of body weight showed that 1) before training, the bodyweight of the SHRs in the five groups showed no significant difference (*p* > 0.05). 2) After training, the bodyweight of the five groups significantly increased (*p* < 0.05) (Figure 2A).

The results of echocardiography showed that 1) before training, EF and FS in hypertension groups were significantly lower than those in WKY (*p* < 0.05), and there was no difference among hypertension groups. 2) After training, EF and FS in the BFRT, LIRT, and HIRT groups significantly increased (*p* < 0.05); EF and FS in the BFRT, LIRT, and HIRT groups significantly increased compared with the SHR-SED, but there was no significant difference in cardiac function among the three groups (*p* > 0.05) (Figure 2B).

Analysis of BP and HR showed that 1) before training, BP and HR in hypertension groups were significantly higher than those in WKY (*p* < 0.05), and there was no significant difference among hypertension groups. 2) After training, the BP and HR of BFRT and LIRT groups significantly decreased, while the BP of HIRT and the DBP of SHR-SED significantly increased (*p* < 0.05). The results of comparison among hypertension groups showed that compared with SHR-SED, the BP and HR of the BFRT and LIRT groups significantly decreased, the BP of the HIRT was significantly increased (*p* < 0.05), and the BP and HR of the BFRT were lower than those in the LIRT group (*p* < 0.05) (Figure 2C).

### VEGF-Pi3k-Akt-eNOS Proteins Expression in the Myocardium and VEGF Protein Expression in Blood

**FIGURE 3:**
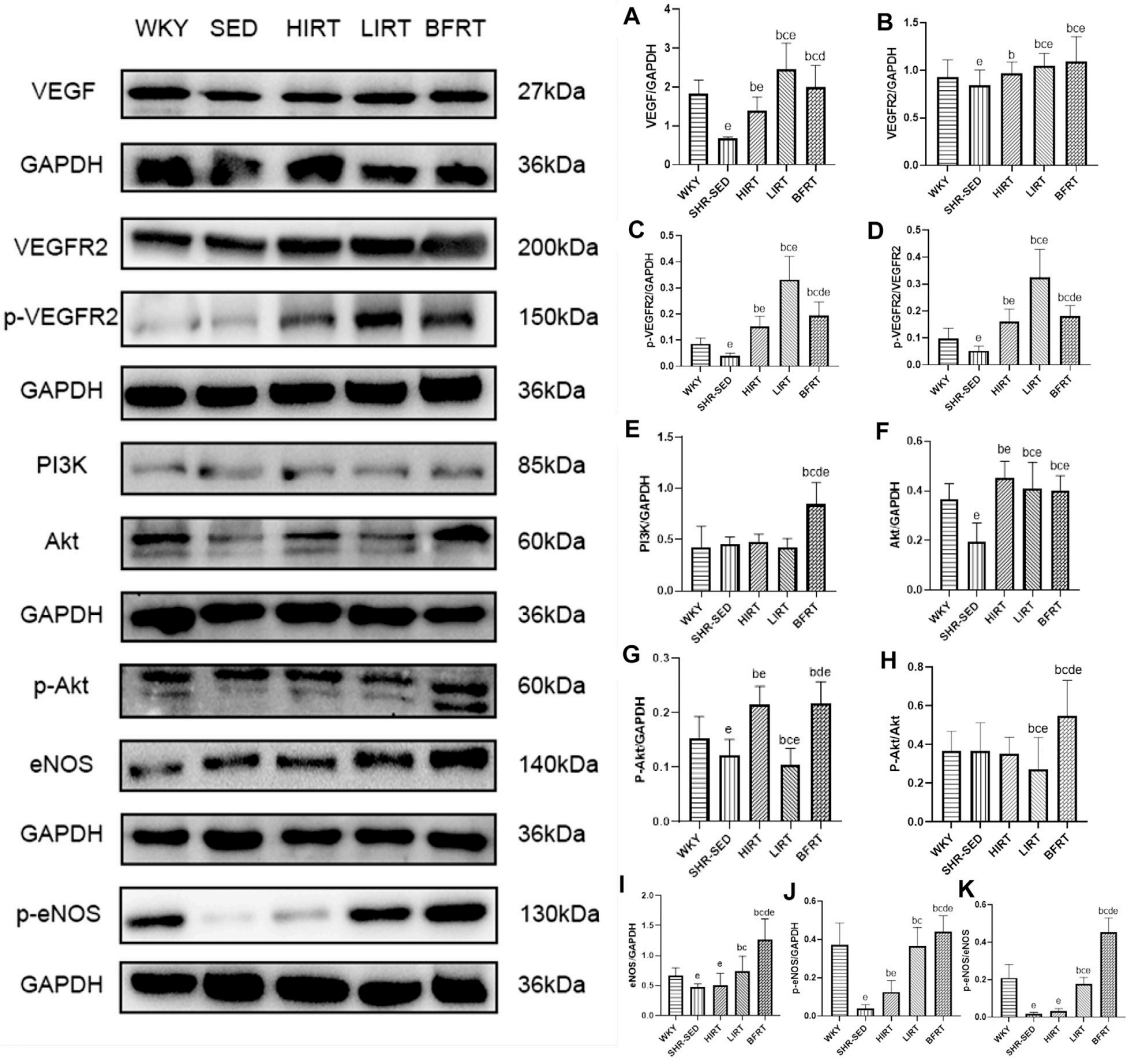
VEGF-Pi3k-Akt-eNOS protein expression in left ventricular tissues. **(A)**, VEGF/GAPDH; **(B)**, VEGFR2/GAPDH; **(C)**, p-VEGFR2/GAPDH; **(D)**, p-VEGFR2/VEGFR2; **(E)**, PI3K/GAPDH; **(F)**, Akt/GAPDH; **(G)**, p-Akt/GAPDH; **(H)**, p-Akt/Akt; **(I)**, eNOS/GAPDH; **(J)**, p-eNOS/GAPDH; **(K)**, p-eNOS/eNOS.

Western blot analysis showed that 1) protein expression of VEGF in the myocardium of the BFRT was significantly upregulated, VEGF after binding to VEGFR2, activation of PI3K, then Akt and then eNOS; In the exercise groups, protein expression of eNOS and p-eNOS in the myocardium of BFRT was the highest, and the ratio of p-eNOS/eNOS in the myocardium of BFRT was significantly higher than that in LIRT and HIRT (*p* < 0.05). 2) Protein expression of VEGF in the myocardium of the LIRT was significantly upregulated, VEGF after binding to VEGFR2, other pathways were activated, then activation of eNOS; In the exercise groups, protein expression of VEGF, VEGFR2 and p-VEGFR2 in the myocardium of the LIRT was the highest, and the ratio of p-VEGFR2/VEGFR2 in the myocardium of the LIRT was significantly higher than that in BFRT and HIRT (*p* < 0.05). 3) Compared with SHR-SED, protein expression of VEGF, VEGFR2 and p-VEGFR2 in the myocardium of the HIRT was significantly upregulated, and the ratio of p-VEGFR2/VEGFR2 in the myocardium of the HIRT was significantly higher than that in SHR-SED (*p* < 0.05) (Figure 3).

**FIGURE 4:**
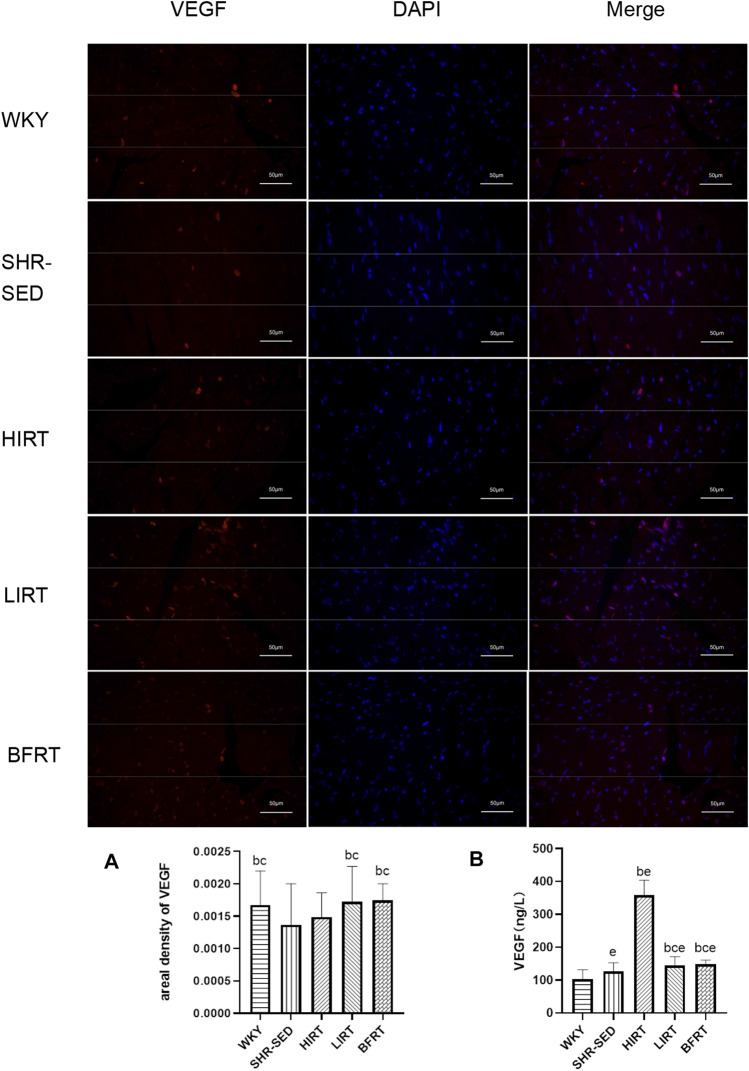
**(A)** Immunofluorescence findings of VEGF in left ventricular tissues. Nucleus is blue by labeling with DAPI. Positive cells are red according to the fluorescent labels used. All the pictures were taken at ×200 magnification, Scale bars, 50 μm. The greater the areal density value, the higher the VEGF fluorescence intensity. **(B)** VEGF protein expression in blood.

ELISA revealed that compared with SHR-SED, the expression of VEGF in blood in the BFRT, HIRT, and LIRT groups was significantly upregulated. Compared with the LIRT and BFRT groups, the upregulation of VEGF in the blood of the HIRT was the highest (*p* < 0.05) (Figure 4B).

### Immunohistochemistry and Immunofluorescence Findings

**FIGURE 5:**
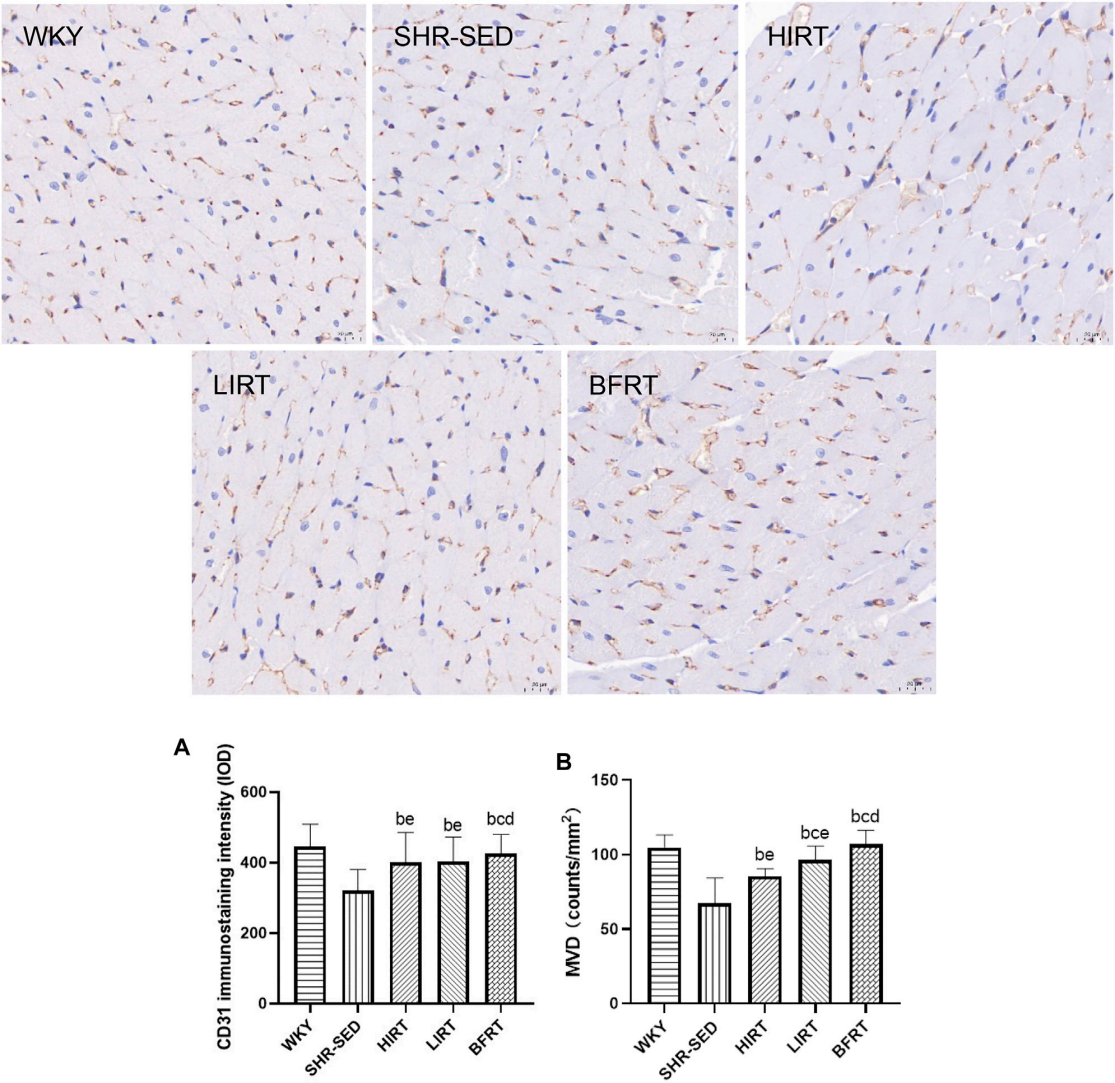
Immunohistochemical staining of PECAM-1/CD31 in left ventricular tissues. All the pictures were taken at ×400 magnification, Scale bars, 20 μm **(A)**, CD31 immunostaining intensity; **(B)** microvessel density (MVD) of myocardium.

Immunohistochemistry findings showed that 1) compared with SHR-SED, optical intensity value of CD31, which means the expressed level of CD31, was increased significantly in WKY, HIRT, LIRT and BFRT groups; compared with WKY and BFRT groups, optical intensity value of CD31 was decreased significantly in HIRT and LIRT groups (*p* < 0.05). 2) Compared with SHR-SED, the capillary density was increased significantly in WKY, HIRT, LIRT, and BFRT groups. Besides, the results showed that the effects of interventions on capillary density in descending order were BFRT > LIRT > HIRT, and difference between each two groups had a statistical significance (*p* < 0.05) (Figure 5).

Immunofluorescence staining of VEGF showed that the level of VEGF in the myocardium of LIRT and BFRT was significantly higher than that of SHR-SED and HIRT (*p* < 0.05), but there was no significant difference among LIRT, BFRT, and WKY (Figure 5).

### Data Correlation Analysis

**FIGURE 6:**
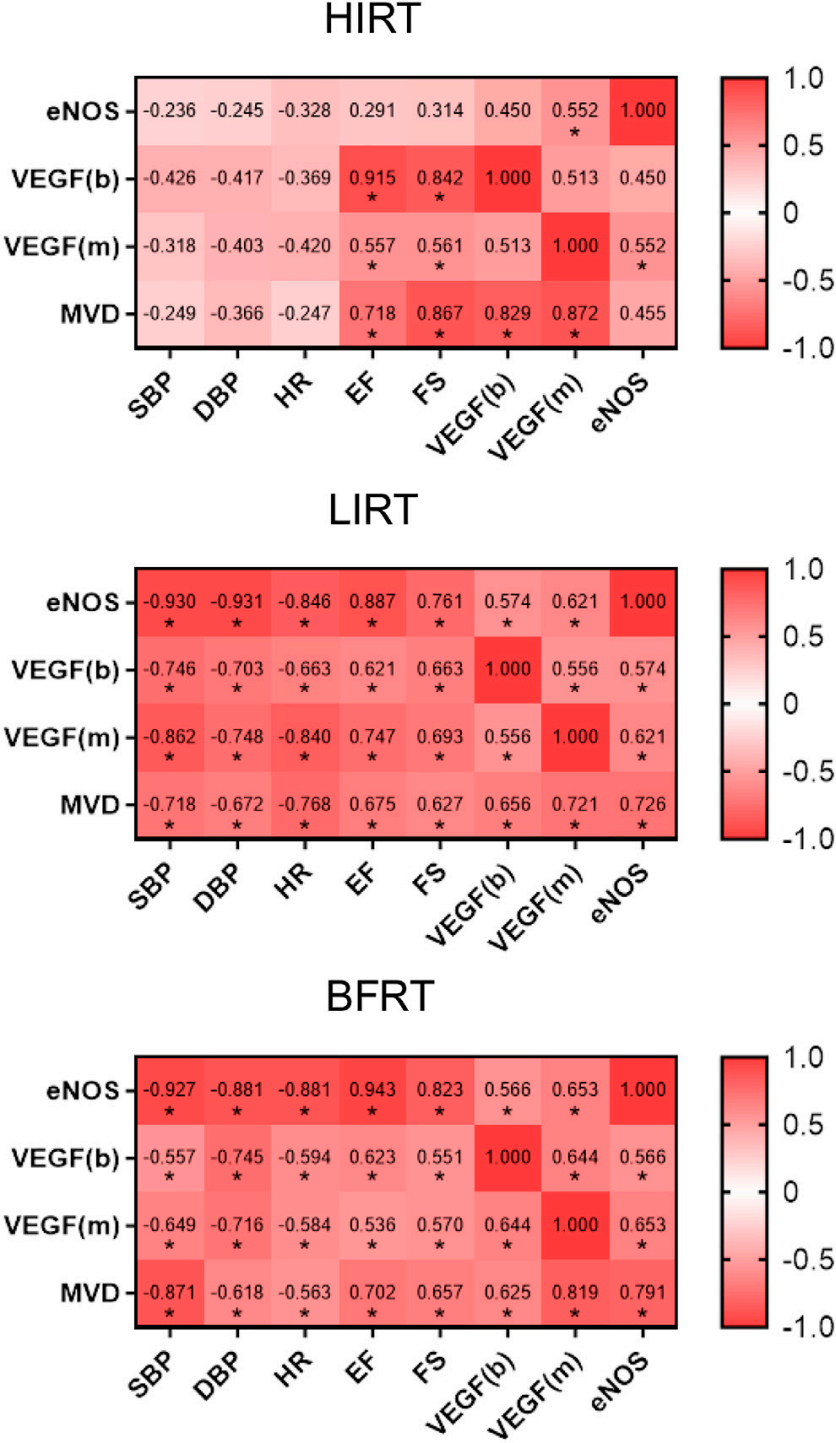
Data correlation analysis.

The results of correlation analysis showed that 1) in the HIRT, VEGF of blood, VEGF of myocardium and MVD were positively correlated with EF and FS, and VEGF of blood and VEGF of myocardium were positively correlated with MVD (*p* < 0.05); 2) in the LIRT and BFRT groups, eNOS, VEGF of blood, VEGF of myocardium, and MVD were negatively correlated with BP and HR, positively correlated with EF and FS (*p* < 0.05), and VEGF of blood, VEGF of myocardium and eNOS were positively correlated with MVD (*p* < 0.05) (Figure 6).

## Discussion

This study was designed to determine and compare the influence of blood flow-restricted low resistance training along with traditional resistance training on microvascular circulation in the myocardia of the SHRs.

In terms of the VEGF-Pi3k-Akt-eNOS pathway, BFRT activated VEGF-Pi3k-Akt-eNOS pathway, VEGF combined with VEGFR2, and then activated eNOS to induce eNOS phosphorylation. At the same time, BFRT promoted the expression of VEGF in blood. It has been reported that when vascular peripheral shear stress increases, the expression of eNOS in endothelial cells is active; this change is due to the increase in blood flow required by dynamic exercise to meet the oxygen supply requirements of the body. After detection of shear stress by receptors on the surface of endothelial cells, a large amount of eNOS is released to ensure that endothelial cells in the state of functional hyperemia can work normally (Casey et al., 2017; Park et al., 2019). VEGF-Pi3k-Akt-eNOS pathway activation can improve endothelial dysfunction and restore cardiac diastolic capacity (Wang et al., 2010; Couto et al., 2018; Palabiyik et al., 2019), but it has also been reported that different exercise contents will have different regulations on the expression of PI3K pathway signal protein even if the exercise type and exercise time are similar (Song et al., 2015). The difference between BFRT and LIRT lay in the limitation of blood flow. Based on the original dynamic exercise, BFRT compressed the blood vessels through cerclage, temporarily reduced the blood flow and limited the blood flow again. After the cerclage was relieved, the blood vessels in the blood flow restricted area were congested for a period of time. This process increased the shear stress of blood vessels and endothelial cells, further stimulated the activation of eNOS and increased the expression of eNOS (Leung et al., 2008; Chantler and Frisbee, 2015; Tegtbur et al., 2020). At the same time, the expression of eNOS in BFRT myocardium was the highest in the exercise groups, and the phosphorylation level of eNOS was the highest. When SHR myocardium in BFRT was exposed to VEGF, the tissue could respond more effectively to the presence of VEGF to support NO production by endothelial capillary which has strong protective effect (Chen et al., 2020). The expression and phosphorylation level of VEGF protein in LIRT myocardium were significantly upregulated after exercise, which was consistent with the results of previous studies (Naderi-boldaji et al., 2018). For HIRT, although it is reported that high-intensity exercise can improve cardiac function, some studies have shown that high-intensity exercise will increase the level of oxidative stress, aggravate endothelial dysfunction, stimulate the increase of ET-1 and inhibit the expression of eNOS. Such changes will affect endothelial function (Novoa et al., 2017; Wang et al., 2019). At the same time, the correlation analysis also showed that the correlation between VEGF in myocardium and VEGF in blood was not statistically significant. In this regard, we believe that HIRT cannot activate the VEGF-Pi3k-Akt-eNOS pathway, and there are other ways to increase VEGF expression in the HIRT myocardium. The increase of VEGF expression in blood comes from other tissues.

In terms of microvascular circulation, as the control group, SHR-SED had the problem of myocardial microvascular rarefaction. BFRT, LIRT, and HIRT could improve myocardial microvascular rarefaction. The capillary density of BFRT was significantly higher than that of traditional resistance training. It has been reported that after exercise, the increase in eNOS and VEGF content helps new capillaries participate in cardiac microvascular circulation, and capillary density can also reflect the response of microvascular circulation, and these changes improve cardiac function, reduce load, and lower blood pressure (Hesketh et al., 2019; D Amarillo, 2019), the same was true of capillary density in exercise groups. According to existing results, the reason why capillary density in BFRT were significantly higher than that in LIRT and HIRT may be that the content of eNOS in BFRT was significantly higher than in LIRT. However, while the capillary density of HIRT improved, the expression of VEGF in blood was abnormally upregulated. Some studies have shown that under high-load endurance exercise and resistance training, VEGF expression increased to repair damages caused by reactive oxygen species (ROS) in the body, and the dynamic balance of microvessels can well express the state between endothelial cells and surrounding fibrotic cells (Item et al., 2013; Brandt et al., 2019). This explains why the expression of VEGF in blood was much higher than that in other groups, but the number of capillary density was lower than that in other groups. We speculate that the improvement of myocardial microvascular circulation and cardiac function in HIRT is due to the upregulation of VEGF, but the capillary density is lower than that in other groups because HIRT does not activate eNOS, and the targeting of VEGF expression may be biased towards ROS injury caused by exercise.

In addition, in terms of cardiac function and blood pressure, BFRT can effectively improve the cardiac function of hypertensive rats, and compared with traditional resistance training, BFRT has a better antihypertensive effect. While HIRT improved cardiac function, it raised blood pressure. For the results of BFRT, previous studies have proved that the acute responses of blood pressure and cardiac function to BFRT in hypertensive elderly women, BFRT can immediately lower blood pressure and improve cardiac function, but it lacks the effect of long-term BFRT on hypertension (Pinto and Polito, 2016; Barili et al., 2018; Pinto et al., 2018). This study verified long-term BFRT can effectively lower blood pressure and improve cardiac function, and the antihypertensive effect is better than the traditional low-intensity resistance training. At the same time, correlation analysis showed that MVD was negatively correlated with blood pressure and heart rate. Combined with relevant research conclusions, after taking part in some exercises, the tachycardia of SHR returns to normal, and the cardiac output and blood pressure of SHR decrease. Therefore, we speculate that BFRT can improve cardiac function and reduce heart rate by improving myocardial microvascular circulation. At the same time, it can still meet the blood supply demand of SHR. When the cardiac output of SHR decreases, the amount of blood flowing into the artery per unit time decreases, the pressure on the arterial wall decreases. Mourad et al. also believe that there is a certain relationship between blood pressure change and myocardial microvascular circulation (Rizzoni et al., 2003; Kaul and Ito, 2004; Mourad and Laville, 2006; Safar and Lacolley, 2007; Rossoni et al., 2011; Ladlow et al., 2018). We also believe that although blood pressure is mainly determined by blood characteristics and total peripheral resistance, there is a correlation between blood pressure and myocardial microvascular circulation. The results of LIRT are consistent with the research conclusions of Neto et al., traditional low-intensity resistance training can effectively lower blood pressure and improve cardiac function (Neto et al., 2015). HIRT results are consistent with the research results of Soares et al., high intensity training has a positive effect on cardiac function (Soares et al., 2019). At the same time, some studies have shown that high-intensity training can improve sympathetic nerve excitability and cause adverse fluctuations of blood pressure (Ozaki et al., 2013; Shimojo et al., 2015). When more than 65%1RM resistance training is carried out, oxidative stress levels increase, endothelial dysfunction is aggravated, large fluctuations in blood pressure occur that lead to adverse blood pressure reactions (Sharman et al., 2015), which also explains why HIRT exhibited elevated blood pressure in the experiment.

In conclusion, blood flow-restricted low resistance training can activate the VEGF-Pi3k-Akt-eNOS pathway, upregulate the expression of VEGF and eNOS, promote myocardial microvascular circulation, improve cardiac function, lower blood pressure and achieve the preventive effect of early hypertension, and the hypotensive effect is better than traditional resistance training.

## Data Availability

The original contributions presented in the study are included in the article/Supplementary Material, further inquiries can be directed to the corresponding author.
